# TSPO Expression Modulatory Effect of Acetylcholinesterase Inhibitor in the Ischemic Stroke Rat Model

**DOI:** 10.3390/cells10061350

**Published:** 2021-05-29

**Authors:** Yoo Sung Song, Sang Hee Lee, Jae Ho Jung, In Ho Song, Hyun Soo Park, Byung Seok Moon, Sang Eun Kim, Byung Chul Lee

**Affiliations:** 1 Department of Nuclear Medicine, Seoul National University Bundang Hospital, Seoul National University College of Medicine, Seongnam 13620, Korea; yosung99@hanmail.net (Y.S.S.); kkpling@snu.ac.kr (S.H.L.); jjh@bioimaging.co.kr (J.H.J.); inosong@gmail.com (I.H.S.); hyuns@snu.ac.kr (H.S.P.); 2 Department of Transdisciplinary Studies, Graduate School of Convergence Science and Technology, Seoul National University, Seoul 08826, Korea; 3 Department of Nuclear Medicine, Ewha Womans University Seoul Hospital, Ewha Womans University College of Medicine, Seoul 07804, Korea; bsmoon@ewha.ac.kr; 4 Department of Molecular Medicine and Biopharmaceutical Sciences, Graduate School of Convergence Science and Technology, Seoul National University, Seoul 08826, Korea; 5 Center for Nanomolecular Imaging and Innovative Drug Development, Advanced Institutes of Convergence Technology, Suwon 16229, Korea

**Keywords:** translocator protein 18-kDa, acetylcholinesterase, neuroinflammation, positron emission tomography, middle cerebral artery occlusion, radioligands, ischemia

## Abstract

We performed in vivo PET imaging with 3-[^18^F]F-CP118,954 (**1**) for acetylcholinesterase (AChE) and [^18^F]fluoromethyl-PBR28-*d*_2_ (**2**) for translocator protein 18-kDa (TSPO) to investigate the inflammatory brain response after stroke. Imaging studies were performed in the middle cerebral artery occlusion (MCAO) Sprague-Dawley rat model for a period of three weeks. The percentage injected dose per tissue weight (%ID/g) of striatum of **1**, and cortex of **2** were obtained, respectively. To trace the sequential inflammatory responses, AChE imaging of **1** was done on post-MCAO day 2, after giving cold PK-11195 for 1 day, and TSPO imaging of **2** was carried out on post-MCAO day 11, after giving donepezil for 10 days. AChE activity in the MCAO-lesioned side were significantly higher than that of the contralateral side on day one, and TSPO activity was highest on day 11. TSPO inhibitor, PK-11195 did not affect AChE activity on day two, while AChE inhibitor, donepezil significantly lowered TSPO binding on day 12. Our study demonstrates that AChE level is elevated in the early course of brain ischemia as a trigger for the inflammatory response, and TSPO level is elevated persistently throughout the post-ischemic injury in the brain. Also, the AChE inhibitor may be able to inhibit or delay neurotoxic inflammatory responses and serve as a beneficial treatment option.

## 1. Introduction

Ischemic stroke is a sudden neurologic deficit resulting in high mortality and morbidity. Numerous clinical trials are ongoing, in order to improve its outcomes, but still has limited results [1]. One reason for the high mortality is due to the limitation of effective treatment options, which are mainly focused on thrombolysis during the acute stage. Currently, thrombolytic therapy is the most effective treatment in improving outcomes by providing reperfusion to the cerebral tissues before irreversible neuronal death. However, less than 10% of total ischemic stroke patients benefit from thrombolytic therapy, due to the narrow time window of disease progression [2]. Additionally, thrombolytic therapy-related complications, such as intracranial hemorrhage occurs in about 6% [3]. Therefore, numerous studies have applied significant effort in finding new possible therapeutic options other than thrombolysis, such as hypothermia therapy or *N*-methyl-D-aspartate receptor antagonist treatments, but none have been proven to be effective yet [4,5].

Recently, neuroinflammation has gained attention as a potential therapeutic target for the treatment of ischemic stroke. While ischemia-induced neuroinflammation takes an important role in the post-stroke reperfusion injury, it also limits the effect of thrombolytic therapy by activating the inflammatory cascade. This process involves several pathophysiologic mechanisms, such as infiltration of inflammatory cells, cytokine secretion, and migration of adhesion molecules [6,7]. Although various clinical trials have been done with efforts to target neuroinflammation as a novel therapeutic target, the complex pathogenesis has interrupted its success.

There have been several in vivo imaging studies that have investigated the pathophysiology of post-ischemic neuroinflammation, mostly demonstrating the increased expression of the translocator protein 18-kDa (TSPO) [8,9,10,11,12,13]. However, TSPO only reflects microglial activation and does not reflect the whole post-ischemic neuroinflammatory cascade. Acetylcholinesterase (AChE) has recently come into focus as a post-stroke neuroinflammatory factor, which is one of the major components of the cholinergic system. It has been previously suggested that AChE has an apoptotic effect and is activated during the neuronal injury associated with ischemia [14,15,16]. Moreover, nicotinic acetylcholine receptors (nAChRs) are suggested to be overexpressed in microglia [17]. However, there has been no direct evidence whether AChE and microglia can affect each other.

**Figure 1:**
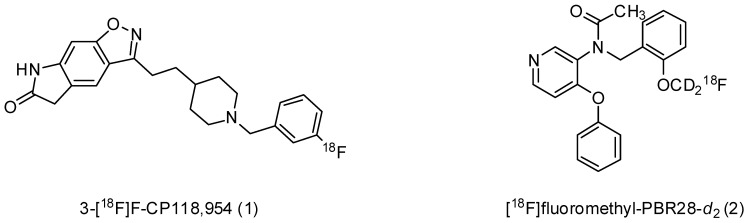
Structures of AChE-binding (**1**) and TSPO-binding (**2**) radioligands.

In the present study, we demonstrate the relationship of microglial activation and AChE activity using TSPO and AChE targeting positron emission tomography (PET) radioligands (Figure 1), and manipulating respective activities with inhibitors, in the ischemic stroke rat model. 

## 2. Materials and Methods

### 2.1. Radiochemical Syntheses of 3-[^18^F]F-CP118,954 (***1***) and [^18^F]Fluoromethyl-PBR28-d_2_ (***2***)

^18^F-Fluoride was produced by ^18^O(p, n)^18^F reaction through proton irradiation using a KOTRON-13 cyclotron at the Seoul National University Bundang Hospital. Radioligands, **1** and **2**, were synthesized as described in the previous studies [18,19].

### 2.2. Ischemic Stroke Rat Model

All animal experiments were approved by Committee of Seoul National University Bundang Hospital (IACUC No. BA1506-179/030-01, approved 19 June 2015). 7-8-week-old male Sprague–Dawley rats were purchased from Orient Bio Inc. (Seoul, Korea) and kept in a specific pathogen-free room maintained at a room temperature of 21 °C and 55% humidity on a 12-h light/dark cycle with food and water available ad libitum. The right middle cerebral artery occlusion (MCAO) surgeries in rats were performed by 60 min occlusion of the right middle cerebral artery, as previously described [20].

### 2.3. Determination of Infarct Volume

After PET imaging study, the rats were anesthetized, then the brains were immediately resected. The resected brains were sectioned into 2 mm coronal sections and immersed in PBS solution (0.1 M) containing 2% of 2-3-5-triphenyltetrazolium chloride purchased from Sigma-Aldrich (St. Louis, MO, USA) at 37 °C for 15 min. The infarct volume was measured by Meta-Morph imaging program (Molecular Devices Inc., Downingtown, PA, USA).

### 2.4. PET Studies

PET studies were performed with a NanoPET/CT system (Mediso Inc., Budapest, Hungary) using the static mode for data acquisition. Rats were anesthetized with 2% isoflurane (7:3 mixture of N_2_/O_2_). After induction of anesthesia, catheters were inserted into the tail vein for intravenous injection of radioligand **1** or **2** targeting AChE or TSPO, respectively. Both PET images were acquired for 20 minutes, from 20 to 40 minutes after injection, in reference to previous studies [18,19]. The apparent behavioral status and vital signs of the subject were monitored during the scan to adjust the anesthesia condition; the temperature of the animals was kept constant at 37 °C. After MCAO surgery, AChE PET images of 3-[^18^F]F-CP118,954 (**1**) were acquired on days 2, 3, 8, 15, and 21 from six rats and TSPO PET images of [^18^F]fluoromethyl-PBR28-*d*_2_ (**2**) were acquired on days 5, 8, 11, 15, and 21 days from five rats. Acquired PET data were reconstructed using iterative 3D–ordered subset expectation maximization, with the single-slice rebinning method. PET images were spatially normalized to the standard stereotaxic space via coregistration to the predefined magnetic resonance imaging (MRI) rat brain template with a comprehensive image processing software (PMOD 3.6, PMOD Group, Geneva, Switzerland). Volume of interests (VOIs) of each striatum and cerebral cortex were drawn manually, for AChE PET and TSPO PET images, respectively. Percentage for injected dose per tissue gram (%ID/g) were acquired from each VOIs. For the PK-11195 and donepezil drug administration study mentioned below, %ID/g ratios of striatum and cortex in the MCAO-lesioned side of brain to that of contralateral areas were used as indices. The statistical significance of differences between groups were tested using the Mann-Whitney U test, with a commercial software package (MedCalc, MedCalc Software v 19.7, Ostend, Belgium).

### 2.5. Drug Administration

**Figure 2:**
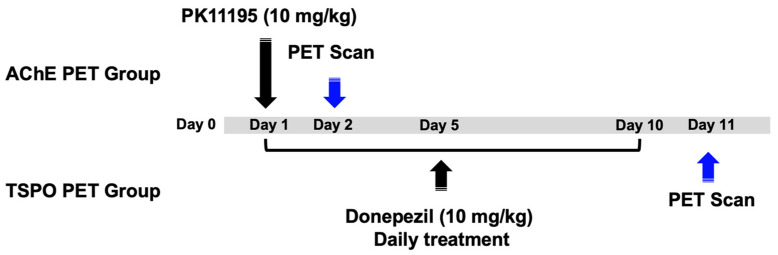
Drug administration and PET studies for the investigation of sequential inflammatory response (each *n* = 6).

For the investigation of sequential inflammatory responses after stroke, PK-11195 was given (10 mg/kg, intravenous administration, *n* = 6 rats) on day 1 after MCAO surgery in one group, and AChE PET images of 3-[^18^F]F-CP118,954 (**1**) were acquired on day 2 after MCAO surgery. Donepezil (10 mg/kg, oral administration, *n* = 6 rats) was daily given for 10 days, the day after MCAO surgery in another group, and TSPO PET images of [^18^F]fluoromethyl-PBR28-*d*_2_ (**2**) were acquired on day 11 after MCAO surgery (Figure 2).

### 2.6. Immunofluorescent Staining

The brain was transcardially perfused with 10% formalin. The isolated brains were embedded in a freezing section compound, frozen and consecutively cryo-sectioned in 10 μm thickness slices using a cryocut microtome (Leica Biosystems). The brain sections were stained with rabbit anti-TSPO antibody or rat anti-CD11b antibody according to the manufacturer’s instructions. After washing with PBS for three times, these sections were incubated with an Alexa 488-conjugated goat anti-rabbit IgG or Alexa 546-conjugated goat anti-rat IgG. All the antibodies were purchased from Thermo Fisher Scientific. Slides were mounted with Slow Fade Gold antifade reagent with 4′,6-diamidino-2-phenylindol (DAPI). Fluorescent images were acquired using a confocal microscope (LSM 710, Zeiss, Oberkochen, Germany).

## 3. Results

### 3.1. Determination of Infarct Volume

**Figure 3:**
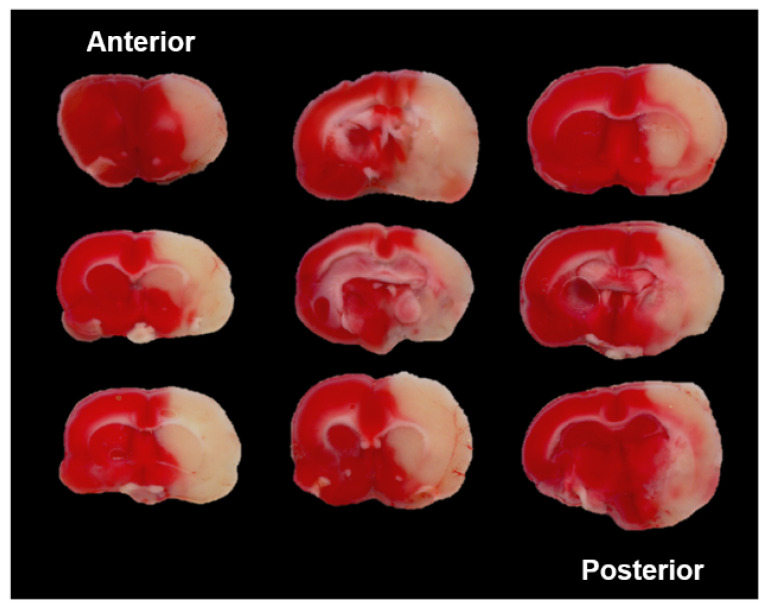
Coronal section images of TTC staining of a MCAO rat brain. Infarct areas (MCAO-lesioned side of the brain) were stained as white.

Triphenyltetrazolium chloride (TTC) staining of rat brain was conducted to validate the MCAO surgery at day 11 and measured the infarct volume (Figure 3). Infarct volume of the right-side cerebral hemisphere was dominant compared with the contralateral left side cerebral hemisphere. The infarct volume measured by TTC stained area was 407.2 ± 39.5 mm^3^ in the MCAO-lesioned side of rat brain.

### 3.2. AChE and TSPO PET Imaging Studies

**Figure 4:**
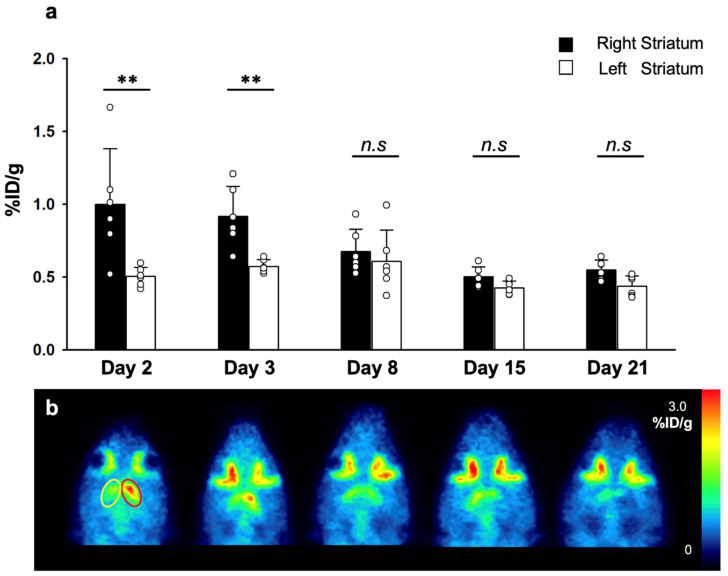
%ID/g values of 3-[^18^F]F-CP118,954 (**1**) on days 2, 3, 8, 15 and 21 after MCAO surgery (**a**). Representative 3-[^18^F]F-CP118,954 (**1**) PET images at respective days (**b**). Red oval indicates the MCAO-lesioned VOI, and yellow oval indicates the contralateral VOI. The statistical significance of differences were determined using the Mann-Whitney U test; ** *p* < 0.01. *n.s.*: not significant.

AChE activities of the MCAO models (*n* = 6) were measured via PET images of **1** which were acquired on days 2, 3, 8, 15, and 21 after MCAO surgery (Figure 4). The right striatal %ID/g in the MCAO-lesioned side of brain was significantly higher than that of the left striatum, on post-MCAO surgery days 2 and 3. The differences were more significant on day 2 compared with day 3. In contrast, the left striatal %ID/g showed no significant differences in 21 days.

**Figure 5:**
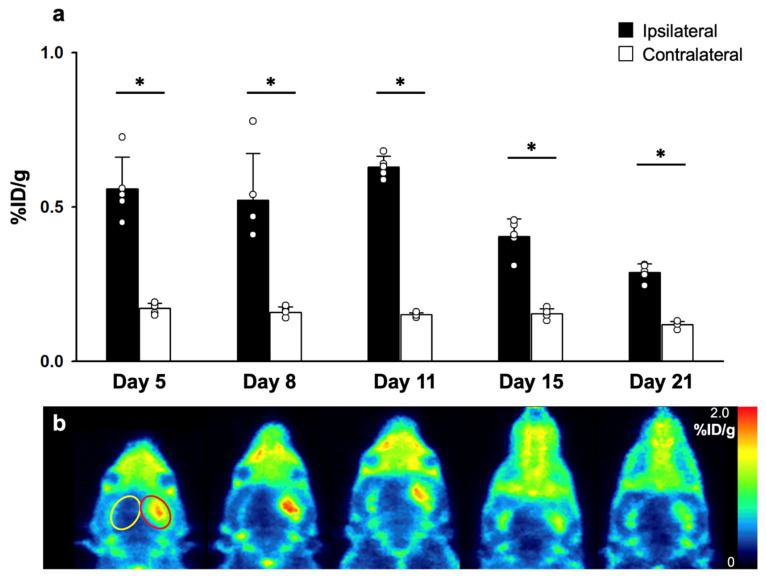
%ID/g values of [^18^F]fluoromethyl-PBR28-*d*_2_ (**2**) on days 5, 8, 11, 15 and 21 after MCAO surgery (**a**). Representative [^18^F]fluoromethyl-PBR28-*d*_2_ (**2**) PET images at respective days (**b**). Red oval indicates the MCAO-lesioned VOI, and yellow oval indicates the contralateral VOI. The statistical significance of differences were determined using the Mann-Whitney U test; * *p* < 0.05.

TSPO activities of the MCAO models (*n* = 5) were measured via PET images of [^18^F]fluoromethyl-PBR28-*d*_2_ (**2**) which were acquired on days 5, 8, 11, 15, and 21 after MCAO surgery (Figure 5). The cerebrocortical %ID/g in the MCAO-lesioned side of the brain was significantly higher than that of the contralateral cerebral cortex, which were maintained from day 5 for a period of 3 weeks. The differences were most significant on day 11. In contrast, the cerebrocortical %ID/g of the contralateral cerebral cortex showed no significant differences for a period of 3 weeks.

### 3.3. AChE and TSPO PET Imaging Study with Treatment of PK-11195 and Donepezil Treatment, Respectively

**Figure 6:**
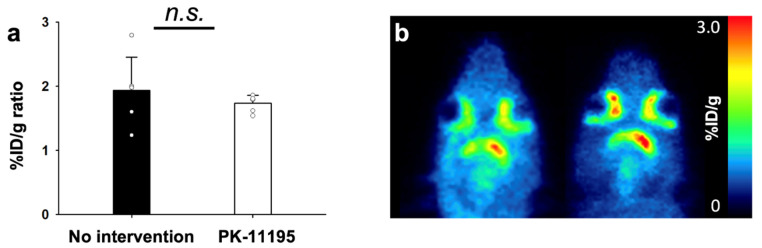
%ID/g ratios of 3-[^18^F]F-CP118,954 (**1**) with and without PK-11195 treatment on day 2 after MCAO surgery (**a**). Representative AChE PET images of respective conditions (**b**). The statistical significance of differences were determined using the Mann-Whitney U test; *n.s.*: not significant.

**Figure 7:**
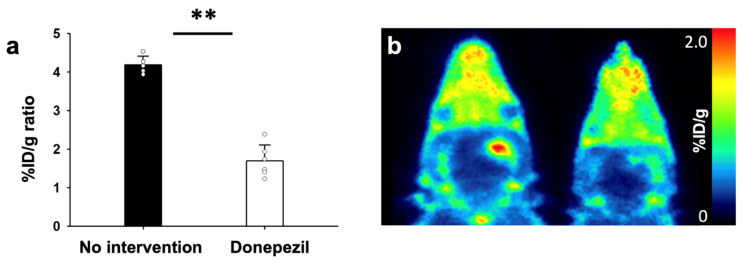
%ID/g ratios of [^18^F]fluoromethyl-PBR28-*d*_2_ (**2**) with, and without, donepezil treatment on day 11 after MCAO surgery (**a**). Representative TSPO PET images of respective conditions (**b**). The statistical significance of differences were determined using the Mann-Whitney U test; ** *p* < 0.01.

**Figure 8:**
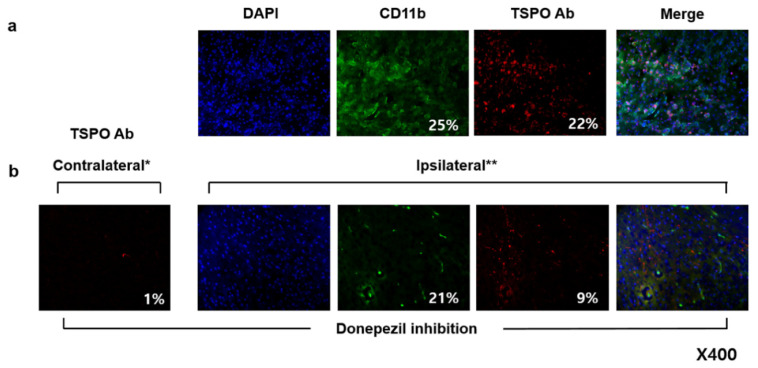
Representative immunohistochemistry image of rat brain on day 11 after MCAO surgery, without any intervention (**a**), and with donepezil treatment for 10 days (**b**). Means % areas of CD11b and TSPO Ab were obtained from multiple animals (*n* = 6). Contralateral *: Non-lesioned side of the brain area. Ipsilateral **: MCAO-lesioned side of the brain area.

Group of rats with and without TSPO blocking treatment (PK-11195) for 1 day underwent AChE PET imaging on day 2 after MCAO surgery (Figure 6). Using PK-11195 treatment (No intervention), uptake values in ipsilateral and contralateral striatum were 1.00 ± 0.38, and 0.50 ± 0.06 %ID/g, respectively. Using PK-11195 treatment, the uptake values in ipsilateral and contralateral striatum were 0.85 ± 0.10 and 0.48 ± 0.06 %ID/g, respectively. Calculated %ID/g ratios of MCAO-sided striatum to the left contralateral striatum for each group indicated that there were no significant differences among the two groups. Group of rats with and without AChE inhibitor treatment (donepezil) for 10 days after MCAO surgery underwent TSPO PET imaging on day 11 after MCAO surgery (Figure 7). Without donepezil treatment, uptake values in ipsilateral and contralateral striatum were 0.63 ± 0.03 and 0.15 ± 0.01 %ID/g, respectively. With donepezil treatment, the uptake values in ipsilateral and contralateral striatum were 0.20 ± 0.09 and 0.12 ± 0.04 %ID/g, respectively. Calculated %ID/g ratios of MCAO-sided cerebral cortex to contralateral cerebral cortex for each group suggest that donepezil significantly decreased the TSPO activity. Immunohistochemistry confirmed PET imaging results, showing decreased expression of TSPO in the rat treated with donepezil, compared with the rat without any intervention in Figure 8.

## 4. Discussion

Post-stroke neuroinflammation has recently gained interest and has been suggested to have potential values as a new therapeutic target, and as a biomarker for one’s prognosis. However, it is a complicated pathophysiology involving various molecular pathways. It is first known to be initiated by the infiltration of immune cells at the site of blood brain barrier (BBB) breakage [21], followed by recruitment, activation and upregulation of various cytokines and inflammatory molecules, in a specific time sequence [22]. Yet, little is known about the role of post-stroke neuroinflammation despite numerous previous studies, and there are uncertainties as to whether it works as a neuroprotective or proinflammatory mechanism. However, neuroinflammation may have a promising future as a prognosis biomarker and a therapeutic target for early stage stroke. This is because the prognosis of stroke is mainly determined during the early stage, while post-stroke neuroinflammation is most active.

During the early hours after the onset of stroke, there are increments of various cytokines such as interleukin-1 beta (IL-1β), interleukin-1 receptor antagonist (IL-1ra), interleukin-6 (IL-6), IL-8, IL-10, and tumor necrosis factor alpha (TNF-α). These cytokines have diverse roles. For instance, IL-1β and TNF-α directly induce the apoptosis of neuronal cells and have a toxic effect, whereas IL-10 suppresses the neurotoxic function of TNF-α [21,22]. This is followed by the activation and recruitment of immune cells, such as the polymorphonuclear leukocytes, macrophages, and microglial cells, which last for several days. Currently, there are only few studies that have demonstrated the cascade of post-stroke neuroinflammation via imaging modalities in human patients. Moreover, among the various neuroinflammatory biomarkers, most of these studies have targeted TSPO, which reflects the microglial activity [9,10,23]. However, the role of microglia in stroke is yet not clear, since it contributes to post-ischemic inflammation by producing proinflammatory cytokines such as TNF and IL-1β, while they also contribute to tissue repair by producing IL-10, tissue growth factor- β (TGF- β), and insulin-like growth factor 1 (IGF-1) [24]. This may be due to the complicated temporal sequences and pathophysiology of microglial activation after stroke. In a previous ischemic stroke model study, microglia expressed high levels of the M2 (anti-inflammatory) at three days and the M1 (proinflammatory) at seven days after stroke [25]. Moreover, remote microglial activity in the brain stem along the affected tract region persisted throughout the recovery period, while microglial activity in the infarct lesion decreased [23]. However, these previous studies have limitations in demonstrating the role and contribution of microglial activation in the post-stroke neuroinflammation cascade. In our study, TSPO activity was significantly decreased by donepezil, a cholinesterase inactivator.

Acetylcholine, which is the major neurotransmitter of the cholinergic neurons, is also known to have a pivotal role in neuroinflammation after stroke [26,27]. In our study, we targeted acetylcholinesterase (AChE) in the reflection of acethylcholine receptor (AChR) activity. After an ischemic event, the cholinergic anti-inflammatory pathway becomes activated by the activation of nicotinic acetylcholine receptors (nAChR), which blocks the production of proinflammatory cytokines, such as TNF-α [28,29]. However, AChE is known to induce apoptosis and form apoptosomes [13], and low AChE level was a significant risk factor reflecting the activation post-stroke cholinergic anti-neuroinflammatory process in a study against stroke patients [29]. The increased expression level of nAChRs on microglia during post-stroke ischemia [17] suggests a missing link between acetylcholine and microglial activation. In our study, we used two radioligands (**1** and **2**) developed by our group, targeting AChE and TSPO, respectively. Microglial activity, assessed by TSPO PET, reached its peak 11 days after MCAO surgery and gradually decreased, but remained significantly increased for three weeks. This pattern of microglial activity in the ischemic stroke rat model is similar with the in vivo PET imaging results of [^18^F]DPA-714 in a rat model of focal cerebral ischemia [13]. AChE activity reached its highest level on day two after MCAO surgery, and returned to normal on day 8. This asymmetric AChE PET in the MCAO-sided striatum and the left contralateral striatum visually reflects the reported time-dependent changes in brain AChE concentrations in MCAO model [14]. Additionally, by administrating donepezil, we demonstrated that the microglial activity was reduced by the inhibition of AChE activity during the early phase of stroke. AChR activation is known to inhibit the production of TNF-α, IL-1, high-mobility group box 1 (HMGB1), the proinflammatory cytokines and molecules, which induces the inflammatory effect of microglias [25,30]. While, AChE activation is suggested to occur earlier than microglial activation in separate studies [13,14], we first demonstrate the direct relationship between AChE and microglia activity. Our study results indicate that acetylcholine starts the neuroinflammatory process, returning to its basal status after triggering microglial activation. Considering the known role of AChR activation mentioned above, we carefully suggest that post-stroke microglial activation acts as a pro-inflammatory process.

AChE inhibitors and TSPO imaging may be suggested as a new therapeutic target and a prognosis biomarker. While tissue plasminogen activator (tPA) is the only effective current therapeutic option for stroke patients, it can only be used at a narrow time window. We expect additive therapeutic effects from donepezil by regulating neuroinflammation in the early phase, while the role of microglial activation still needs further investigation. Previous studies that have investigated the effect of AChE inhibitor after stroke only focused on the improvement of cognitive functions [31,32].

However, there are some limitations in our study. First, due to the complex process and pathways of post-stroke neuroinflammation, we assume that there are more complicated interactions and molecules involved between the AChE and microglial activation, than we suggested. Second, there is currently no established method for the quantification of TSPO-binding radioligands [33]. Here, we used the ratios of %ID/g, which could have been affected by blood flow. Third, our conclusions need statistical evidence of immunohistochemistry data to be identified in future studies.

## 5. Conclusions

In conclusion, our study shows that the post-stroke neuroinflammatory process can be demonstrated with AChE and TSPO PET imaging, and suggests the future possibility of its utilization as a prognostic and therapeutic biomarker.

## Data Availability

All the data presented in this study are included in this article.
